# Functional Trait Variability of *Salicornia europaea* L. Across Inland Saline Habitats: Integrating Shoot and Root Morphometrics with Soil Salinity and Trophic Status

**DOI:** 10.3390/plants14213383

**Published:** 2025-11-05

**Authors:** Agnieszka Piernik, Nour Elhouda Gargouri, Piotr Hulisz, Ahmad Rajabi Dehnavi, Stefany Cárdenas Pérez

**Affiliations:** 1 Department of Geobotany and Landscape Planning, Faculty of Biology and Veterinary Sciences, Nicolaus Copernicus University in Toruń, Lwowska 1, 87-100 Toruń, Poland; gargourinourelhouda@gmail.com (N.E.G.); rajabi@umk.pl (A.R.D.); 2 Department of Soil Science and Landscape Ecology, Faculty of Earth Sciences and Spatial Management, Nicolaus Copernicus University in Toruń, Lwowska 1, 87-100 Toruń, Poland; hulisz@umk.pl

**Keywords:** soil salinity, population, soil-legacy effects, halophyte, soil–plant relationship

## Abstract

*Salicornia europaea* is an extremely salt-tolerant annual halophyte. It occurs in coastal and inland saline habitats and is increasingly cultivated for food, nutraceuticals, and environmental remediation. This study examined whether inland populations from contrasting saline sites exhibit heritable differences in shoot and root morphology. Seeds from four isolated sites (Ciechocinek, Inowrocław, Salzgraben, and Soltauquelle) were grown at 0, 200, 400, and 1000 mM NaCl, and morphometric traits were quantified from digital images. Corresponding soil samples were also analyzed. A strong relationship was found between population origin and responses to salt stress. Optimal growth generally occurred at 200–400 mM NaCl. Shoot canopy area consistently best discriminated among populations. Inowrocław and Salzgraben performed best under extreme salinity (1000 mM), whereas Ciechocinek showed the weakest growth. Root analyses revealed a shift from radial expansion at moderate salinity to elongation at higher levels, with Salzgraben and Soltauquelle maintaining the longest roots. Soil analyses indicated substantial site differences: Ciechocinek had the highest salinity, resulting in the smallest trait development, Inowrocław was rich in Ca^2+^ and organic matter, while the German sites had lower salinity but higher pH and bicarbonates. These findings demonstrate population-specific adaptive strategies and soil-legacy effects, supporting targeted ecotype selection for saline agriculture and phytoremediation.

## 1. Introduction

*Salicornia europaea* L., commonly known as glasswort, sea asparagus, or marsh samphire, is a succulent annual halophyte of the *Amaranthaceae* family (subfamily *Salicornioideae*). As a euhalophyte, it completes its life cycle under saline conditions and is regarded as one of the most salt-tolerant terrestrial plants [1]. Salt tolerance in *S. europaea* is achieved through vacuolar sequestration of Na^+^ via NHX antiporters, selective ion uptake by HKT-type transporters, and accumulation of compatible osmolytes such as proline and glycine betaine [2,3,4]. Its simple highly succulent morphology with photosynthetic activity restricted to segmented green stems and vestigial leaves minimizes transpirational water loss and supports survival in hypersaline habitats [5]. Physiological and biochemical studies further demonstrate that salinity stimulates the accumulation of phenolic compounds, anthocyanins, and β-carotene, which act as antioxidants and enhance nutritional value [6,7,8]. The species develops shallow but extensive fibrous root systems that enable efficient water and ion uptake in waterlogged saline soils, contributing to productivity under brackish and hypersaline conditions [9,10,11].

The natural distribution of *S. europaea* spans European coasts, estuarine mudflats, and inland salt basins, but it has also colonized anthropogenically salinized landscapes, such as post-mining brine ponds, reflecting considerable ecological plasticity [12,13]. Cytogenetic and molecular analyses distinguish diploid *S. europaea* (2*n* = 18) from its polyploid relatives, consolidating its identity within the Eurasian diploid clade [14]. These features, combined with its simple architecture, short life cycle, and uniform growth habit, make it an excellent model species for studying halophyte adaptation. Beyond its ecological role, *S. europaea* has become increasingly relevant in saline agriculture, biotechnology, and remediation. Field trials in Europe and Asia confirm its potential for cultivation under brackish or seawater irrigation, with optimal biomass production at moderate salinity [8,15,16]. Commercial cultivation has been established in Mexico and the USA, where shoots are marketed as gourmet vegetables, while extracts are developed into functional foods, nutraceuticals, cosmetics, and forage products [17,18,19]. At the same time, phytoremediation studies highlight its ability to tolerate and accumulate heavy metals in contaminated saline soils, supporting its use in wastewater treatment and soil reclamation [20,21,22]. Collectively, *S. europaea* represents a multifunctional halophyte at the intersection of ecology, agriculture, and biotechnology.

Investigating this species at the population scale provides insights into local adaptation as distinct habitats exert strong selective pressures on functional traits. Populations are defined as groups of interbreeding individuals sharing a habitat, but their boundaries and divergence often depend on spatial scale and environmental heterogeneity [23]. In halophytes, variation across fine spatial scales may reflect short-term plasticity, while broader geographical separation can result in ecotypic differentiation and genetic divergence [24]. For *S. europaea*, exposure to fluctuating salinity regimes may promote trait plasticity, whereas populations from more stable saline environments often evolve specialized salt tolerance strategies [25]. Functional trait-based approaches are particularly effective for capturing these differences. Traits such as succulence, stem diameter, root length, and tissue dimensions directly reflect fitness under saline conditions, while anatomical modifications, such as lignification and cell wall remodeling, enhance osmotic stability [12,26,27]. Considerable intraspecific variability has been documented among natural populations, underlining the value of trait-based analyses for disentangling plastic versus genetic-based responses to salinity stress.

Based on this framework, the present study tests the hypothesis that inland populations of *S. europaea* from ecologically distinct saline habitats exhibit heritable differences in morphometric traits that reflect both adaptive genetic responses and maternal habitat legacies. We assumed that long-term exposure to divergent salinity regimes has shaped population-specific shoot and root growth strategies, resulting in distinct responses when grown under uniform controlled conditions. Specifically, we assess the shoot and root dimensions and functional traits across four populations subjected to graded NaCl treatments (0, 200, 400, and 1000 mM). By integrating population soil-characteristic origin with salinity effects, this study seeks to clarify the extent of local specialization and plasticity in this model halophyte.

## 2. Results

### 2.1. Shoot Trait Variability

The ANOVA-II results (Table 1) reveal significant effects of population for all the shoot traits (*p* < 0.001) and highly significant population × salinity interactions (*p* < 0.001), indicating that the magnitude and direction of responses differed among the populations. By contrast, the main effect of salinity was not significant for S-Projected area, S-Height, or S-Diameter, but S-Canopy (*p* = 0.022) and S-Shape perimeter (*p* < 0.001) were significantly influenced by NaCl concentrations.

The morphometric analysis of shoots by one-way ANOVA (ANOVA-I; Table 2) confirmed distinct population-specific responses across the salinity treatments. At 0 mM NaCl, representing salt deprivation, growth was generally reduced compared with 200 and 400 mM, with smaller S-Projected areas, shorter S-Height, and restricted S-Canopy development, particularly in the Ciechocinek and Soltauquelle populations. Under non-saline conditions, the populations did not differ in S-Projected area or S-Shape perimeter (Table 2). The Salzgraben population had significantly greater S-Height and S-Diameter, and the Inowrocław population had the largest S-Canopy. Growth improved in all the populations at 200 and 400 mM NaCl, and the differences among them became more pronounced. The C-population consistently exhibited the lowest values across the traits. At 200 mM, the I-population developed the widest S-Canopy but the smallest S-Diameter. The So- and Sa-populations reached the greatest S-Height. At 400 mM, the Sa-population exhibited the widest S-Canopy, followed by the I-population. Moreover, the Sa-, I-, and So-populations showed significantly greater S-Projected area and S-Height compared with the C-population. At 1000 mM, strong population divergence was observed. The I- and Sa-populations performed best, showing significantly higher values across all the shoot traits, followed by the So- and finally C-populations.

**Figure 1:**
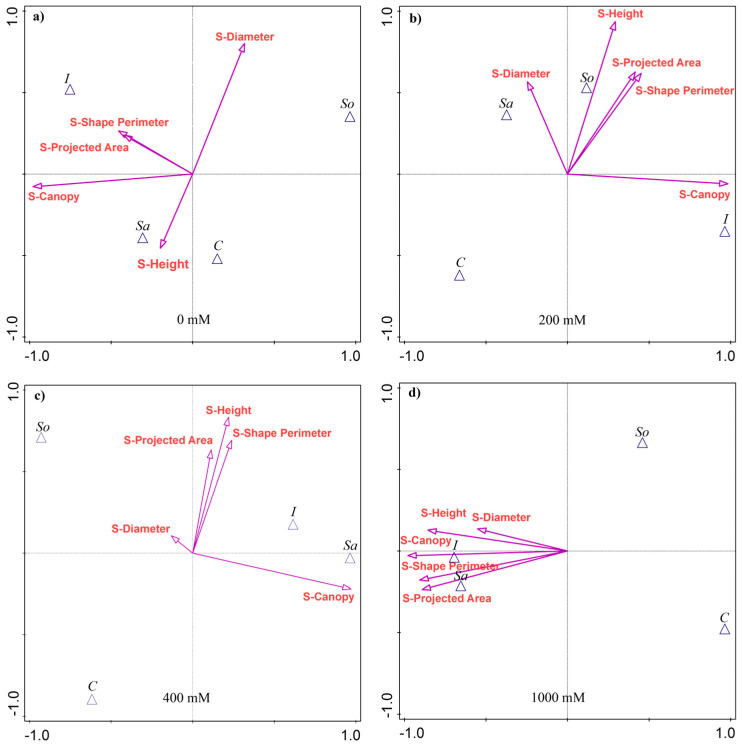
Canonical variate analysis (CVA) of shoot morphological traits in four *Salicornia europaea* populations under different salinity treatments: (**a**) 0 mM NaCl, (**b**) 200 mM NaCl, (**c**) 400 mM NaCl, and (**d**) 1000 mM NaCl. Vectors indicate trait contributions to population discrimination. Population abbreviations: I—Inowrocław, C—Ciechocinek, Sa—Salzgraben, and So—Soltquelle.

The results of the discriminant analysis showed that the shoot traits most separating the populations varied with treatment, although S-Canopy was consistently the strongest discriminator, explaining 22–30% of the variability among the populations (Appendix A Table A1). At 0 mM NaCl, in addition to S-Canopy, S-Diameter explained the most variability, followed by S-Height and S-Shape perimeter. The I- and Sa-populations were associated with larger S-Canopies, while the I- and So-populations were associated with greater S-Diameter (Figure 1a).

At 200 mM, height was the second most discriminant trait (18% of variability), followed by S-Shape perimeter (13.3%) and S-Projected area (12.7%). The I-population was associated with the largest S-Canopy, while Sa- and So-populations were linked to other traits (Figure 1b). At 400 mM, the discriminant traits were the same as at 200 mM, although they explained a smaller share of the variability, except for S-Canopy (Table A1). The Sa- and I-populations were associated with larger S-Canopies, and, together with the So-population, with the remaining traits (Figure 1c). At 1000 mM, S-Canopy accounted for 29.7% of the variability, followed by S-Shape perimeter (26%), S-Projected area (25.3%), S-Height (22.9%), and S-Diameter (10.3%). The Sa- and I-populations were associated with high values across the traits, while the So- and C-populations performed poorly (Figure 1d).

### 2.2. Root Trait Variability

The two-way ANOVA (Table 3) revealed highly significant population effects for all the root traits (*p* < 0.001) and significant population × salinity interactions (*p* < 0.01). In contrast, the main effect of salinity was not significant for root projected area, shape perimeter, or length.

The one-way ANOVA (ANOVA-I; Table 4) confirmed distinct population-specific responses across the salinity treatments. At 0 mM, the I-population displayed significantly lower root values than the others, while the So-population had the longest roots. Similar results were observed at 200 mM (Table 4). At 400 mM, the Sa-, So-, and C-populations performed similarly, whereas I- again showed significantly reduced root traits. At 1000 mM, population divergence was more pronounced: the C-population had the lowest root growth, the I-population maintained reduced root length, and the Sa- and So-populations retained relatively high root development (Table 4).

**Figure 2:**
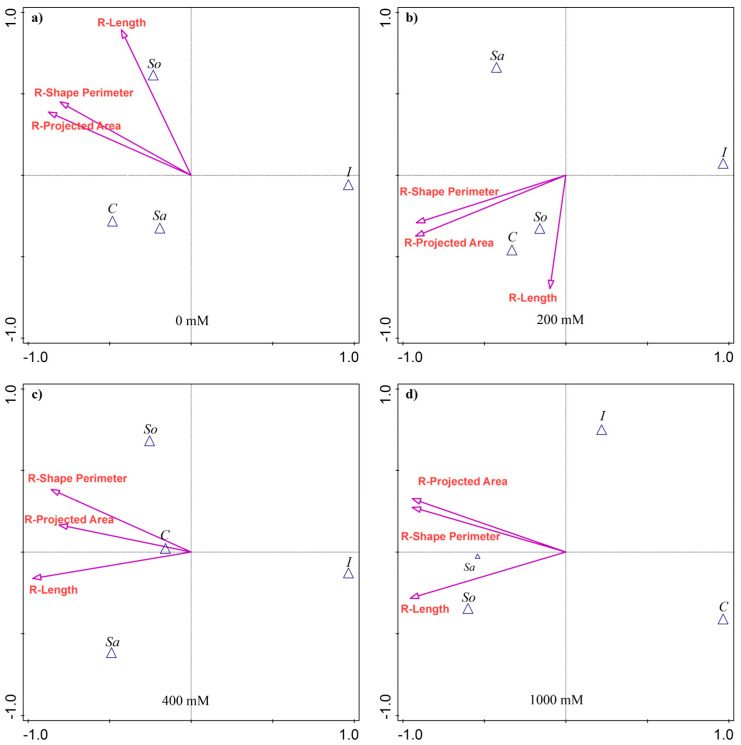
Canonical variate analysis (CVA) of root morphological traits in four *S. europaea* populations under different salinity treatments: (**a**) 0 mM NaCl, (**b**) 200 mM NaCl, (**c**) 400 mM NaCl, and (**d**) 1000 mM NaCl. Vectors indicate trait contributions to population discrimination. Population abbreviations: I—Inowrocław, C—Ciechocinek, Sa—Salzgraben, and So—Soltquelle.

The discriminant analysis (CVA) showed that the root traits most separating the populations varied with treatment (Figure 2). At 0 mM, no traits contributed significantly to population variability. At 200 mM, the R-Projected area (25.7% of the explained variability) and the R-Shape perimeter (25.2%) accounted for most of the differences among the populations (Appendix A Table A2). The So- and C-populations were most associated with greater R-Length (Figure 2b). At 400 mM, R-Length explained the largest share of variation (26.1%), followed by R-Shape perimeter (22.7%), with the C-population relatively more aligned with R-Projected area (Figure 2c). At 1000 mM, all three traits contributed substantially, each explaining about 29% of the variability (Appendix A Table A2). Root R-Length dominated, followed by R-Projected area and R-Shape perimeter. The So-population was associated with greater R-Length, while the Sa-population corresponded more closely with R-Shape perimeter and R-Projected area. The C-population was positioned opposite, reflecting the lowest values for these traits (Figure 2d).

### 2.3. Comparison of Population Growth Performance

**Figure 3:**
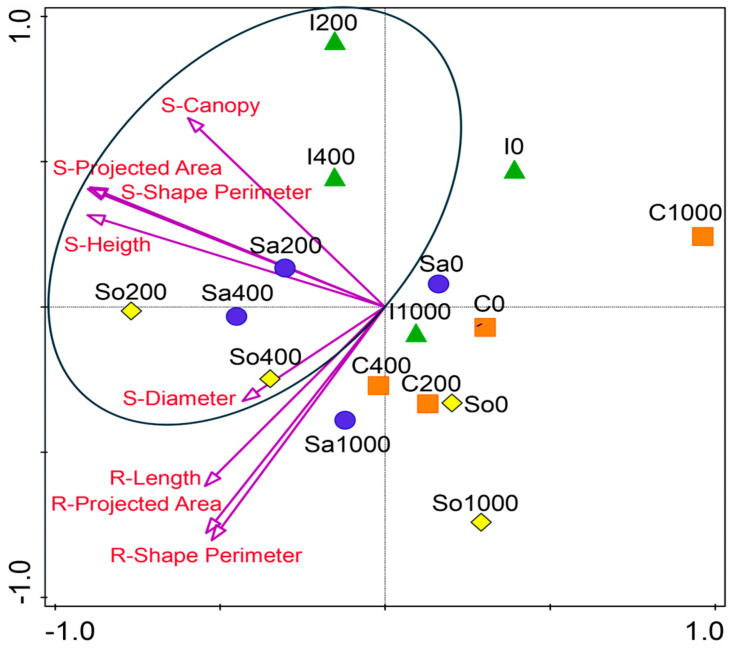
Non-metric multidimensional scaling (NMDS) ordination of standardized shoot and root traits of *Salicornia europaea* populations under four salinity levels (0, 200, 400, and 1000 mM NaCl) based on Euclidean distance. Points represent populations (I—Inowrocław, C—Ciechocinek, Sa—Salzgraben, and So—Soltquelle); vectors indicate shoot (S) and root (R) traits.

The NMDS ordination of standardized shoot and root traits revealed population-specific performance patterns across salinity levels (Figure 3). In most populations, optimum growth occurred at 200 or 400 mM NaCl, indicating that these levels supported the most favorable trait development in *S. europaea*. Populations I200, I400, Sa200, Sa400, So200, and So400 clustered along a shared axis in NMDS space, reflecting strong trait expression and alignment with the shoot and root vectors. In contrast, the C population remained clearly separated from the central cluster, showing the weakest alignment with the shoot traits across the treatments (Figure 3). However, within this population, growth at 200 and 400 mM was relatively improved compared with 0 and 100 mM NaCl.

### 2.4. Differences in Site Environmental Conditions

Soil variables were considered the main factors shaping population differences. The chemical analyses revealed pronounced variation in salinity and edaphic properties among the four inland sites where the *S. europaea* populations were collected (Table 5 and Table 6). For salinity (Table 5), the C-site showed the highest electrical conductivity (48.1 dS·m^−1^) and elevated Na^+^ (9427 mg·dm^−3^) and Cl^−^ (16,990 mg·dm^−3^) concentrations. The I-site had the highest Ca^2+^ content (3578 mg·dm^−3^) and intermediate Na^+^ (3296 mg·dm^−3^). By contrast, the Sa- and So-sites had lower salinity, with mean EC_e_ values of 12.7 and 11.0 dS·m^−1^, respectively, and comparatively lower ion concentrations.

For trophic status (Table 6), the I-site had the highest total organic carbon (4.96%), total nitrogen (0.50%), and CaCO_3_ (29.5%), whereas the C-site had the lowest TOC (1.30%) and TN (0.15%). The Sa- and So-sites showed intermediate TOC and TN values, with So distinguished by the highest available phosphorus (Pca, 1628 mg·kg^−1^). The soil pH was slightly alkaline across all the sites, ranging from 7.3 to 8.5 in H_2_O and 7.7 to 8.3 in KCl.

**Figure 4:**
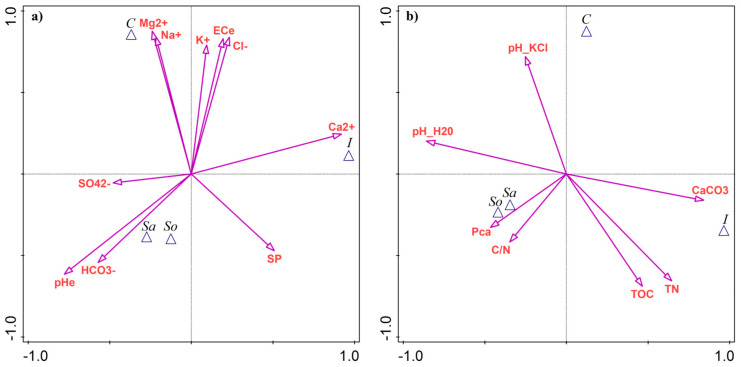
Results of canonical variate analysis (CVA) as a discriminant analysis of soil parameters between four *Salicornia europaea* sites: (**a**) salinity-related variables; (**b**) edaphic parameters. Site abbreviations: C—Ciechocinek, I—Inowrocław, Sa—Salzgraben, and So—Soltauquelle. Soil parameters: pH_e_—pH of saturated paste extract, EC_e_—electrical conductivity of saturated paste extract, SP—saturation percentage, TN—total nitrogen, TOC—total organic carbon, and Pca—plant-available phosphorus.

Based on CVA with forward selection and Monte Carlo permutation testing, soil pH (pH_e_) explained the largest proportion of variance between the sites (32.8%, Appendix A Table A3). The Sa- and So-sites had the highest pH_e_ values (Figure 4a). Calcium (Ca^2+^) was the second most discriminating variable, contributing 30.6% of the explained variance, with elevated levels in the I-site (Figure 4a). The highest total salinity, expressed as EC_e_ and ion contents (Mg^2+^, Na^+^, K^+^, and Cl^−^), was associated with the C-site, explaining 21–27% of the variability between sites (Table A3). Bicarbonates (HCO_3_^−^) explained 21.0% of the variance and were linked with the Sa- and So-sites (Figure 4a).

Only four edaphic parameters contributed significantly to site separation, each explaining ~25% of the variance. Soil pH in water (pH-H_2_O) was followed by CaCO_3_, total nitrogen, and total carbon (Table A3). The Sa- and So-sites were associated with pH-H_2_O, while the I-site was linked with the remaining variables (Figure 4b).

## 3. Discussion

This study demonstrates that inland populations of *S. europaea* exhibit distinct above- and below-ground functional strategies when exposed to salinity gradients under controlled growth chamber conditions. The significant population and population × salinity effects revealed by ANOVA confirm that the shoot and root traits diverged consistently across the stress levels, indicating that intraspecific variability reflects phenotypic plasticity and population-specific differentiation. Such differentiation is increasingly recognized as a key driver of ecological stability in inland halophyte systems [5,27,28].

### 3.1. Aboveground Responses

The most responsive shoot trait was the S-Projected area, followed by S-Height, S-diameter, S-Shape perimeter, and S-Canopy. The populations differed in their strategies of canopy development under stress. Despite modest root investment, Inowrocław maintained strong canopy expansion (high S-Projected area and S-Shape perimeter), suggesting a strategy of prioritizing photosynthetic surface and carbon gain under salinity. Salzgraben sustained S-Projected area and R-Length, reflecting a balanced growth strategy across the compartments. Soltauquelle expressed high S-Height with long roots at moderate salinity, but this coordination declined at 1000 mM, indicating allocation trade-offs at extreme stress levels. In contrast, Ciechocinek consistently displayed low shoot trait values across the treatments, indicative of restricted tolerance thresholds and early growth limitations. These patterns align with previous observations in halophytes, where vertical elongation or canopy expansion serve as a key adaptive mechanism to sustain water uptake and photosynthetic efficiency under moderate salinity but are compromised under extreme ionic stress [1,29].

### 3.2. Belowground Responses

R-Projected area, R-Shape perimeter, and R-Length emerged as critical discriminants of tolerance strategies. The divergence across the populations reflected a shift from radial expansion (R-Projected area and R-Shape perimeter) under moderate salinity to axial elongation (R-Length) under higher stress. This is consistent with the findings regarding *Suaeda salsa* and *Tamarix* spp., where radial thickening initially enhances water and ion buffering, while elongation later supports deeper soil exploration under severe salt stress [30,31]. Salzgraben and Soltauquelle maintained higher R-Length under moderate and high salinity, whereas Inowrocław displayed consistently reduced root elongation, confirming compartment-specific trade-offs. These results reinforce the idea that belowground plasticity provides critical buffering capacity for aboveground growth but varies strongly with population origin.

### 3.3. Soil–Trait Linkages

Although the plants were grown under standardized conditions, the maternal-site soil analyses provided important ecological context. Ciechocinek, enriched in Na^+^ and Cl^−^ with very high EC_e_, produced offspring with limited S- and R-traits across the treatments, reflecting inherited constraints under ionic toxicity. Inowrocław, originating from Ca^2^- and organic matter-rich soils, expressed strong S-Projected area and S-Shape perimeter, consistent with calcium’s role in maintaining membrane stability and Na^+^/K^+^ homeostasis [32]. Salzgraben displayed sustained R-Length and S-Projected area, and Soltauquelle showed high S-Height and R-Length at moderate salinity, highlighting the role of buffering capacity of carbonate soil and phosphorus availability in energy metabolism and osmotic adjustment [33]. The CVA ordinations confirmed these associations, indicating that morphometric responses under controlled salinity align with site-specific edaphic profiles. This suggests that populations retain an ecological “memory” of their soil environments, expressed through coordinated S–R trait combinations.

### 3.4. Applied Significance

From an applied perspective, population-specific strategies highlight the importance of selecting appropriate ecotypes for ecological restoration of inland salt marshes, breeding programs targeting biofunctional traits, or industrial uses such as biofuel and nutraceutical production. For example, populations investing in canopy expansion (Inowrocław, with high S-Projected area and S-Shape perimeter) may be more suitable for biomass applications, whereas those maintaining deeper root elongation (Salzgraben and Soltauquelle: high R-Length) may be better candidates for soil stabilization and remediation purposes. Furthermore, the link between soil ionic profiles and plant functional traits underscores the need to consider site edaphic history when designing restoration or cultivation programs.

### 3.5. Integration with Halophyte Ecology

These findings support the broader concept that halophyte populations adapt through general plasticity and site-specific selective pressures, resulting in ecotypic divergence [12,24]. In *S. europaea*, this divergence is expressed as distinct shoot–root trait combinations: canopy-dominant (Inowrocław: strong S-Projected area and S-Shape perimeter), balanced (Salzgraben: sustained S-Projected area and R-Length), vertically elongated (Soltauquelle: high S-Height and R-Length), and dwarf conservative (Ciechocinek: low S- and R-traits).

These patterns are summarized in Figure 3, which integrates the shoot, root, and soil parameters across the populations. The conceptual model illustrates that Ciechocinek is constrained by high-Na^+^/Cl^−^ soils, leading to poor canopy and short roots; Inowrocław benefits from Ca^2+^-rich soils, sustaining canopy expansion despite modest root investment. Salzgraben and Soltauquelle, associated with carbonate-rich soils and enriched in available phosphorus (Pca), maintain balanced shoot–root elongation under moderate salinity. The figure underscores that populations retain ecological memory of their maternal soils, expressed as contrasting functional strategies under controlled salinity gradients. Such strategies mirror the patterns described in other halophytes (*Atriplex*, *Tripolium*, and *Suaeda*), where divergence across populations enhances community-level resilience under fluctuating saline conditions [5,29].

### 3.6. Limitations and Perspectives

While the controlled chamber approach enabled direct testing of salinity gradients, it also restricted root exploration and microbial interactions, which are central in field conditions. Future studies should integrate physiological and biochemical measurements (ion accumulation, osmotic adjustment, and stress markers) and exploit non-destructive technologies, such as computer vision systems (CVSs) and hyperspectral imaging, for real-time monitoring of plant performance [34]. Combining morphometric, physiological, and omics approaches will enable a deeper mechanistic understanding of how *S. europaea* populations integrate soil legacy with functional trait responses.

## 4. Materials and Methods

### 4.1. Field Work

**Figure 5:**
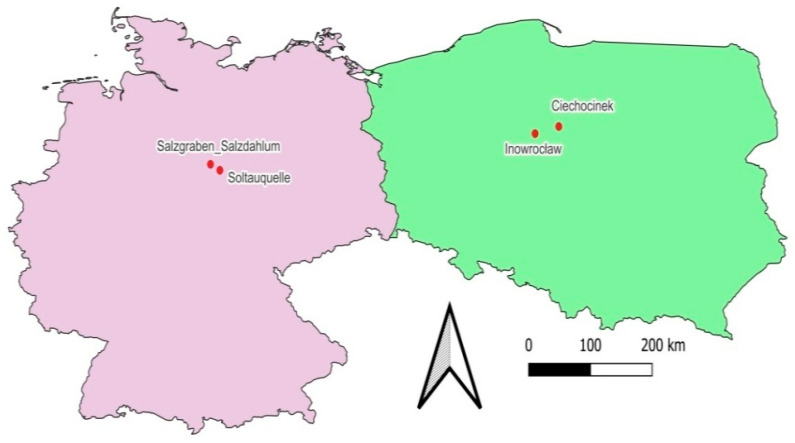
Sampling locations in Germany and Poland.

Seeds of *S. europaea* were collected in late autumn 2022 from four isolated populations (Figure 5): two in Poland—Ciechocinek (52°53′20.3″ N 18°47′06.4″ E; C-population) and Inowrocław (52°45′35.2″ N 18°13′39.9″ E; I population), sampled in November, and two in Germany—Salzgraben–Salzdahlum (52°11′56.8″ N 10°36′05.3″ E; Sa-population) and Soltauquelle (52°05′23.7″ N 10°49′17.9″ E; So-population), sampled in December. According to the Köppen–Geiger classification [35], the study areas in Poland and Germany have a warm-temperate fully humid climate with warm summers (Cfb). The mean annual air temperature and precipitation for the German sites are 9.9 °C and 614.8 mm, respectively (Braunschweig station, 1991–2020; http://sklima.de; accessed on 24 October 2025). For the Polish sites, the corresponding values are 8.9 °C and 548.8 mm (Toruń station, 1991–2020; https://klimat.imgw.pl; accessed on 26 October 2025). Sampling points were located in *Salicornietum ramossissimae* association [36]. In each site, 30 random individuals of *S. europaea* containing seeds were taken and then dried for seed collection. The C-population was located in the nature reserve protecting natural inland salt marshes, which was established in 1964 [37]. Due to the historical drainage of the Vistula Valley, halophytic vegetation is now restricted to a ditch system crossing the reserve. It is not regularly supported by brine from the graduation towers nearby, but the recorded soil salinity is very high, amounting to about 100 dS·m^−1^ [38]. The I-population originates from the Inowrocław–Mątwy industrial saline site located adjacent to the soda factory and affected by the saline waste of soda production, resulting in relatively stable and very high soil salinity around 55 dS·m^−1^ [28,39]. The Sa-population was collected from Salzgraben–Salzdahlum, a naturally inland saline wetland in northern Germany. This habitat is characterized by stable groundwater discharge with long-term consistent salinity exposure [12]. The So-population was gathered from Soltauquelle, a spring-fed inland wetland in northern Germany with shallow calcareous soils. This site experiences strong seasonal shifts in groundwater flow, leading to highly variable salinity conditions [12]. For soil characteristics, four soil samples were taken from the root zone (0–25 cm) for laboratory analyses at each site.

### 4.2. Pot Experiment

Seeds of *S. europaea* were germinated in Petri dishes on filter paper moistened with distilled water. After germination, seedlings approximately 5 mm long were transplanted into individual plastic pots (5.3 cm height, 5.5 cm diameter, and 85.7 cm^3^ volume) filled with a 1:2 mixture of vermiculite and quartz sand. Before planting, nine pots per treatment and population were placed in cuvettes that lacked drainage and were watered to full substrate capacity with relevant solutions of 0, 200, 400, or 1000 mM NaCl. Initially, each pot contained 3–4 seedlings of the respective population, and, later, they were thinned to one plant per pot.

For the first 20 days after transplantation, pots were irrigated every three days with 300 cm^3^ of distilled water adjusted to the assigned NaCl concentration. Thereafter, irrigation continued every three days with 250 cm^3^ of modified Hoagland’s nutrient solution of the same parameters for each salt treatment. The stock solutions were prepared as follows: 236.1 g·dm^−3^ Ca(NO_3_)_2_·4H_2_O, 101.1 g·dm^−3^ KNO_3_, 136.1 g·dm^−3^ KH_2_PO_4_, and 246.5 g·dm^−3^ MgSO_4_·7H_2_O. Trace elements were prepared per 1 dm3 as 2.8 g H_3_BO_3_, 1.8 g MnCl_2_·4H_2_O, 0.2 g ZnSO_4_·7H_2_O, 0.025 g H_2_MoO_4_, and 0.1 g CuSO_4_·5H_2_O. Iron was supplied as Fe-EDTA, obtained by mixing 10.4 g EDTA·2Na and 7.8 g FeSO_4_·7H_2_O in 56.1 g KOH solution, with pH adjusted to 5.5 before mixing. The final Hoagland working solution per 1 dm^3^ contained 7 cm^3^ Ca(NO_3_)_2_, 5 cm^3^ KNO_3_, 2 cm^3^ KH_2_PO_4_, 2 cm^3^ MgSO_4_, 1 cm^3^ trace elements, and 1 cm^3^ Fe-EDTA solution. Plants were maintained under controlled growth chamber conditions: 25 °C day temperature, 50–60% relative humidity, and a 16 h light/8 h dark photoperiod.

### 4.3. Morphometric Plant Measurements

To quantify the morphological variation of *S. europaea* across salinity treatments and populations, all shoot and root traits were measured using digital image analysis. Each shoot was imaged in two orthogonal orientations, front (side-view) and top-view, against a uniform white background with a calibrated metric scale. Images were processed in Fiji (ImageJ v1.53c) using a standardized workflow to segment the shoot silhouette and extract morphometric traits [40,41]. The S-Projected area was defined as the two-dimensional area of the aboveground shoot silhouette captured in a standardized side-view image. S-Canopy referred to the planform area of the shoot obtained from a top-view image. S-Shape perimeter was the total length of the shoot silhouette contour measured on the segmented image. S-Height was the vertical distance from the stem base to the apical tip, and S-Diameter was the maximum width orthogonal to shoot height. For each population and NaCl treatment (0, 200, 400, and 1000 mM), nine shoots (*n* = 9 per population × treatment) were imaged under identical conditions, and all metrics were computed from the resulting binary masks in calibrated units.

Root traits comprised R-Projected area (cm^2^), R-Shape perimeter (cm), and R-Length (cm). Immediately after harvesting and gentle rinsing, roots were photographed in planar (front) view against a matte black background with an embedded scale bar. Images were analyzed in Fiji using the same segmentation workflow as for shoots. R-Projected area represents the total two-dimensional area of the root silhouette, R-Shape perimeter corresponds to the external contour of the whole root, and R-Length denotes the longest visible axis across the captured root system. A total of three root systems per population per salinity treatment (*n* = 3) were analyzed.

### 4.4. Soil Analyses

The soil samples were air-dried, passed through a 2 mm mesh sieve to remove large debris, and homogenized. The contents of total carbon (TC) and total nitrogen (TN) were determined using a Vario Macro Cube CHN/CHNS elemental analyzer (Elementar Analysensysteme GmbH, Langenselbold, Germany). The calcium carbonate (CaCO_3_) content was measured volumetrically using a Scheibler apparatus following the procedure described by Pansu and Gautheyrou [42]. Total inorganic carbon (TIC) was calculated from the CaCO_3_ content, and total organic carbon (TOC) was obtained as the difference between TC and TIC. Soil pH was measured potentiometrically with a CP-551 pH meter (Elmetron, Poland) in both a distilled water suspension (pH-H_2_O) and a 1 M KCl solution (pH-KCl) at a soil-to-solution ratio of 1:2.5. The plant-available phosphorus (Pca) content was determined colorimetrically using a UV-1601 spectrophotometer (Rayleigh, China) after extraction with ammonium citrate solution [43].

Analyses of the saturated paste extract were conducted using the methodology described by van Reeuwijk [43]. Portions of air-dried soil were placed into 250 cm^3^ beakers to about half their volume (≈200 g) and mixed with redistilled water added incrementally until a soil paste was obtained. The beakers were covered and left overnight. The paste was then vacuum-filtered on a Büchner funnel using a vacuum pump. The resulting saturated paste extract was used for further analysis. The extract pH (pHe) was measured potentiometrically using a CP-551 pH meter (Elmetron). Electrical conductivity at 25 °C (EC_e_) was determined by the conductometric method with a CPC-401 conductivity meter (Elmetron). Saturation percentage (SP) was determined gravimetrically by oven-drying. Magnesium (Mg^2+^) and calcium (Ca^2+^) were measured by atomic absorption spectrometry (AAS), whereas potassium (K^+^) and sodium (Na^+^) were determined by flame emission spectrometry (FES) using a SOLAAR Unicam 969 flame spectrometer (Unicam Ltd.,Cambridge, UK). Bicarbonate (HCO_3_^−^) was determined by titration following Pokojska [44]. Sulfate (SO_4_^2−^) and chloride (Cl^−^) were measured by ion chromatography using a Thermo Scientific Dionex Aquion system (Thermo Scientific, Waltham, MA, USA).

### 4.5. Statistical Analysis

We evaluated morphological responses of *S. europaea* across four populations (Inowrocław, Ciechocinek, Saltzgrabe, and Soltquelle) subjected to four salinity levels (0, 200, 400, and 1000 mM NaCl) in a fully crossed two-factor design. Two-way ANOVA (ANOVA-II) tested the effects of population, salinity, and their interaction on morphological traits. Normality was checked with the Shapiro–Wilk test and homogeneity of variance by Levene’s test (*p* < 0.05). Normally distributed traits were further analyzed using one-way ANOVA (ANOVA-I) with Tukey’s HSD post hoc test, applied per trait and salinity level. The Kruskal–Wallis test with Dunn’s post hoc comparisons was used for skewed traits. Analyses were performed in PAST v.5.2.1 [45].

Canonical variate analysis (CVA) assessed trait contributions to population differentiation under each treatment, using forward selection and Monte Carlo permutation tests [46,47]. The simple effects model, focusing on independent trait contributions, was interpreted. All CVA analyses were run in Canoco v.5.

Morphometric differences across salinity treatments were summarized with non-metric multidimensional scaling (NMDS) using Euclidean distance [47]. Before analysis, shoot and root data were z-standardized (mean = 0, SD = 1). Analyses were based on population means per salinity level, with trait vectors overlaid to show direction and magnitude of influence.

Soil parameters were grouped into salinity-related (EC_e_, Na^+^, K^+^, Ca^2+^, Mg^2+^, Cl^−^, SO_4_^2−^, and HCO_3_^−^) and edaphic variables (TOC, TN, C/N, CaCO_3_, P_2_O_5_, Pca, pH-H_2_O, and pH-KCl). Differences between sites were tested with the Kruskal–Wallis test and Dunn’s post hoc comparisons (PAST v.5.2.1). To evaluate variable contributions to site separation, CVA with forward selection and Monte Carlo permutation tests were applied to both soil groups in Canoco v.5.

## 5. Conclusions

We found a strong relationship between population origin and responses to salt stress. Shoot canopy area consistently best discriminated among populations, and the I- and Sa-populations performed best under extreme salinity (1000 mM), whereas the C-population showed the weakest growth. The root analyses revealed a shift from radial expansion at moderate salinity to elongation at higher levels, with the Sa- and So-populations maintaining the longest roots. The soil analyses indicated substantial site differences: the C-site had the highest salinity, resulting in the smallest plant trait development, the I-site was rich in Ca^2+^ and organic matter, while the Sa- and So-sites had lower salinity but higher pH and bicarbonates. These findings demonstrate population-specific adaptive strategies and soil-legacy effects, supporting targeted ecotype selection for saline agriculture and phytoremediation.

## Figures and Tables

**Table 1:** Two-way ANOVA results for shoot morphometric traits of four *S. europaea* populations across salinity treatments. Values represent F-statistics with associated *p*-values; * indicates significance levels (*p* < 0.05) and ** (*p* < 0.01), while NS indicates not significant.

Trait	Population	Population × Salinity	Salinity
S-Projected Area	313.00 **	10.14 **	2.18 ^NS^
S-Canopy	1955.19 **	111.58 **	3.91 *
S-Height	764.41 **	16.40 **	0.28 ^NS^
S-Shape Perimeter	1516.15 **	12.56 **	11.34 *
S-Shoot Diameter	1258.90 **	10.69 **	0.40 ^NS^

**Table 2:** Morphometric shoot traits of four *S. europaea* inland populations (C—Ciechocinek, I—Inowrocław, Sa—Salzgraben, and So—Soltauquelle) grown under different salinity levels (0, 200, 400, and 1000 mM NaCl). Values represent mean ± SE (*n* = 9). Different letters within each salinity treatment indicate significant differences between populations according to ANOVA followed by post hoc Tukey’s HSD test (*p* < 0.05).

Population-Salinity	S-Projected Area	S-Height	S-Diameter	S-Shape Perimeter	S-Canopy
C0	81.19 ± 7.17 ^a^	7.75 ± 0.41 ^b^	0.17 ± 0.01 ^b^	2336.67 ± 83.87 ^a^	108.91 ± 7.53 ^b^
I0	110.29 ± 8.74 ^a^	7.65 ± 0.38 ^b^	0.20 ± 0.01 ^a^	2748.00 ± 105.22 ^a^	139.52 ± 3.39 ^a^
Sa0	107.57 ± 10.14 ^a^	9.62 ± 0.59 ^a^	0.18 ± 0.01 ^b^	2693.56 ± 143.27 ^a^	122.66 ± 4.61 ^a^
So0	86.77 ± 9.31 ^a^	7.54 ± 0.67 ^b^	0.23 ± 0.02 ^a^	2408.89 ± 122.82 ^a^	72.55 ± 7.16 ^c^
C200	90.59 ± 8.29 ^c^	6.52 ± 0.46 ^c^	0.24 ± 0.01 ^a^	2473.33 ± 117.67 ^b^	126.13 ± 7.18 ^c^
I200	181.16 ± 28.99 ^ab^	10.69 ± 1.11 ^b^	0.22 ± 0.01 ^b^	3446.89 ± 266.46 ^a^	279.08 ± 13.15 ^a^
Sa200	162.41 ± 23.31 ^b^	12.34 ± 0.93 ^a^	0.28 ± 0.02 ^a^	3229.33 ± 230.02 ^a^	149.97 ± 5.60 ^c^
So200	217.44 ± 16.53 ^a^	14.09 ± 0.86 ^a^	0.29 ± 0.02 ^a^	3719.33 ± 154.53 ^a^	192.94 ± 1.42 ^b^
C400	106.77 ± 8.60 ^b^	8.06 ± 0.58 ^b^	0.28 ± 0.02 ^a^	2631.33 ± 77.49 ^b^	129.37 ± 3.67 ^c^
I400	155.48 ± 9.79 ^a^	12.19 ± 0.72 ^a^	0.28 ± 0.02 ^a^	3200.22 ± 94.32 ^a^	208.09 ± 5.01 ^b^
Sa400	149.99 ± 22.23 ^a^	10.84 ± 0.42 ^a^	0.28 ± 0.01 ^a^	3226.00 ± 202.55 ^a^	236.09 ± 10.77 ^a^
So400	164.83 ± 15.70 ^a^	11.80 ± 0.88 ^a^	0.29 ± 0.01 ^a^	3241.56 ± 146.94 ^a^	78.22 ± 3.47 ^d^
C1000	42.89 ± 4.50 ^b^	3.22 ± 0.32 ^c^	0.25 ± 0.01 ^c^	1675.33 ± 82.96 ^c^	43.48 ± 2.14 ^c^
I1000	117.24 ± 9.93 ^a^	8.27 ± 0.72 ^a^	0.32 ± 0.01 ^a^	2754.00 ± 128.50 ^a^	92.82 ± 2.87 ^a^
Sa1000	121.76 ± 8.31 ^a^	8.18 ± 0.51 ^a^	0.31 ± 0.02 ^a^	2864.89 ± 93.88 ^a^	90.60 ± 3.49 ^a^
So1000	53.51 ± 4.00 ^b^	5.23 ± 0.37 ^b^	0.28 ± 0.01 ^b^	1893.33 ± 77.21 ^b^	57.29 ± 1.65 ^b^

**Table 3:** Two-way ANOVA results for root morphometric traits of four *S. europaea* populations under different salinity treatments. Values represent F-statistics with associated *p*-values; ** indicates significance levels (*p* < 0.01), while NS indicates not significant.

Trait	Population	Salinity	Population × Salinity
R-Projected area	11.51 **	1.58 ^NS^	3.70 **
R-Shape perimeter	12.52 **	1.41 ^NS^	2.99 **
R-Length	9.94 **	1.44 ^NS^	3.66 **

**Table 4:** Morphometric root traits of four *Salicornia europaea* inland populations (C—Ciechocinek, I—Inowrocław, Sa—Salzgraben, and So—Soltauquelle) grown under different salinity levels (0, 200, 400, and 1000 mM NaCl). Values represent mean ± SE (*n* = 3). Different letters within each salinity treatment indicate significant differences between populations according to ANOVA followed by post hoc Tukey’s HSD test (*p* < 0.05).

Population-Salinity	R-Projected Area	R-Shape Perimeter	R-Length
C0	264.31 ± 13.15 ^a^	4154.67 ± 114.11 ^a^	20.61 ± 2.18 ^ab^
I0	227.07 ± 18.62 ^b^	3872.00 ± 178.95 ^b^	18.73 ± 2.17 ^b^
Sa0	259.92 ± 8.99 ^a^	4136.00 ± 68.35 ^a^	20.14 ± 1.13 ^ab^
So0	270.96 ± 5.31 ^a^	4240.00 ± 39.46 ^a^	24.08 ± 2.56 ^a^
C200	277.20 ± 8.94 ^a^	4240.00 ± 71.11 ^a^	24.43 ± 1.56 ^b^
I200	227.01 ± 11.29 ^b^	3813.33 ± 119.14 ^b^	21.62 ± 7.45 ^b^
Sa200	266.07 ± 3.49 ^a^	4192.00 ± 33.31 ^a^	18.71 ± 1.55 ^c^
So200	273.27 ± 4.32 ^a^	4264.00 ± 36.95 ^a^	31.29 ± 1.16 ^a^
C400	267.80 ± 11.13 ^a^	4213.33 ± 67.30 ^a^	25.65 ± 2.40 ^a^
I400	247.59 ± 7.51 ^b^	4010.67 ± 70.70 ^b^	17.02 ± 0.57 ^b^
Sa400	273.52 ± 1.09 ^a^	4232.00 ± 0.00 ^a^	28.83 ± 1.47 ^a^
So400	274.16 ± 2.75 ^a^	4293.33 ± 32.44 ^a^	25.38 ± 1.52 ^a^
C1000	218.90 ± 4.17 ^b^	3802.67 ± 55.10 ^b^	13.55 ± 1.48 ^b^
I1000	256.03 ± 6.69 ^a^	4093.33 ± 62.03 ^a^	18.33 ± 1.07 ^b^
Sa1000	270.13 ± 5.85 ^a^	4208.00 ± 39.46 ^a^	29.21 ± 0.74 ^a^
So1000	270.65 ± 4.67 ^a^	4234.67 ± 20.83 ^a^	31.81 ± 3.18 ^a^

**Table 5:** Soil salinity characteristics of four inland sites (C—Ciechocinek, I—Inowrocław, Sa—Salzgraben, and So—Soltauquelle) where *Salicornia europaea* populations were collected. Values represent mean ± standard deviation (SD, *n* = 3). Different letters within each salinity treatment indicate significant differences between sites according to the Kruskal–Wallis test followed by post hoc Dunn’s test (*p* < 0.05).

	Site			
Soil Salinity Characteristics	C	I	Sa	So
pH_e_	7.5 ± 0.1 ^b^	7.1 ± 0.0 ^c^	8.1 ± 0.1 ^a^	8.0 ± 0.1 ^a^
EC_e_ [dS m^−1^]	48.1 ± 11.4 ^a^	36.8 ± 6.24 ^a^	12.7 ± 11.9 ^b^	11.0 ± 10.4 ^b^
SP [%]	39.9 ± 7.9 ^a^	79.6 ± 12.7 ^a^	65.9 ± 20.1 ^a^	65.7 ± 18.1 ^a^
Ca^2+^ [mg dm^−3^]	659± 195 ^b^	3578 ± 1019 ^a^	244 ± 131 ^b^	220 ± 124 ^b^
Mg^2+^ [mg dm^−3^]	300.7 ± 85.2 ^a^	82.5 ± 31.8 ^b^	52.3 ± 56.2 ^b^	40.0 ± 42.0 ^b^
Na^+^ [mg dm^−3^]	9427 ± 2238 ^a^	3296 ± 454 ^b^	2291 ± 2301 ^b^	2011 ± 2044 ^b^
K^+^ [mg dm^−3^]	156 ± 43.7 ^a^	99.8 ± 68.9 ^a^	38.1 ± 30.3 ^b^	41.3 ± 28.6 ^b^
HCO_3_^−^ [mg dm^−3^]	419 ± 112 ^a^	320 ± 148 ^b^	629 ± 91.2 ^a^	564 ± 89.2 ^a^
SO_4_^2−^ [mg dm^−3^]	433 ± 103 ^a^	129 ± 30.7 ^a^	470 ± 363 ^a^	389 ± 325 ^a^
Cl^−^ [mg dm^−3^]	16,990 ± 5434 ^a^	12,106 ± 2575 ^a^	3630 ± 4585 ^b^	2958 ± 3782 ^b^

**Table 6:** Soil trophic characteristics of four inland sites (C—Ciechocinek, I—Inowrocław, Sa—Salzgraben, and So—Soltauquelle) where *Salicornia europaea* populations were collected. Values represent mean ± standard deviation (SD, *n* = 3) for total organic carbon (TOC), total nitrogen (TN), C/N ratio, soil pH (in H_2_O and KCl), calcium carbonate (CaCO_3_), and available phosphorus (Pca). Different letters within each salinity treatment indicate significant differences between sites according to the Kruskal–Wallis test followed by post hoc Dunn’s test (*p* < 0.05).

Site	TOC [%]	TN [%]	C/N	pH-H_2_O	pH-KCl	CaCO_3_ [%]	Pca [mg·kg^−1^]
C	1.30 ± 0.64 ^b^	0.15 ± 0.09 ^b^	9 ± 1 ^a^	8.1 ± 0.2 ^a^	8.1 ± 0.2 ^a^	14.2 ± 3.52 ^b^	365 ± 162 ^a^
I	4.96 ± 1.04 ^a^	0.50 ± 0.09 ^a^	10 ± 1 ^a^	7.5 ± 0.2 ^b^	7.8 ± 0.1 ^a^	29.5 ± 2.45 ^a^	387 ± 171 ^a^
Sa	3.55 ± 1.48 ^a^	0.26 ± 0.08 ^b^	12± 2 ^a^	8.2 ± 0.2 ^a^	7.9 ± 0.2 ^a^	8.75 ± 5.09 ^b^	1352 ± 1027 ^a^
So	2.66 ± 1.15 ^a^	0.25 ± 0.08 ^b^	11 ± 2 ^a^	8.2 ± 0.2 ^a^	7.9 ± 0.1 ^a^	11.1 ± 7.82 ^b^	1628 ± 1526 ^a^

## Data Availability

Data is contained within the article.

## Appendix A

Supplementary data to the main text.

**Table A1:** Results of canonical variate analysis (CVA) forward selection and Monte Carlo permutation test of shoot morphological trait effects in the discrimination between *Salicornia europaea* populations in different salt treatments (0, 200, 400, and 1000 mM NaCl). NS—not significant.

Trait	% Variance Explained	*p*	% Variance Explained	*p*	% Variance Explained	*p*	% Variance Explained	*p*
	0 mM		200 mM		400 mM		1000 mM	
S-Canopy	22.8	0.002	28.9	0.002	30.7	0.002	29.7	0.002
S-Diameter	10.6	0.01	7.9	0.032	0.8	NS	10.3	0.008
S-Height	8.3	0.026	18.8	0.002	14	0.002	22.9	0.002
S-Shape Perimeter	7.5	0.036	13.3	0.002	9.9	0.008	26	0.002
S-Projected Area	6.7	NS	12.7	0.004	7.1	0.04	25.3	0.002

**Table A2:** Results of canonical variate analysis (CVA) forward selection and Monte Carlo permutation test of shoot morphological trait effects in the discrimination between *Salicornia europaea* populations in different salt treatments (0, 200, 400, and 1000 mM NaCl).

Trait	% Variance Explained	*p*	% Variance Explained	*p*	% Variance Explained	*p*	% Variance Explained	*p*
	0 mM		200 mM		400 mM		1000 mM	
R-Projected area	15.9	NS	25.7	0.014	18.4	NS	29.4	0.004
R-Length	14.2	NS	13.8	NS	26.1	0.008	29.7	0.002
R-Shape perimeter	10.3	NS	25.2	0.014	22.7	0.016	29	0.004

**Table A3:** Results of forward selection and Monte Carlo permutation test of canonical variate analysis (CVA)—simple effects of soil salinity parameters and soil edaphic conditions in the discrimination between *S. europaea* sites. pH_e_—pH of saturated paste extract, EC_e_—electrical conductivity of saturated paste extract, SP—saturation percentage, TN—total nitrogen, TOC—total organic carbon, and P_ca_—plant-available phosphorus.

	Soil Salinity		Soil Edaphic Condition		
Variable	Explains %	*p*	Variable	Explains %	*p*
pH_e_	32.8	0.002	pH-H_2_O	25.3	0.002
Ca^2+^	30.6	0.002	CaCO_3_	24.7	0.01
Mg^2+^	27.4	0.006	TN	24.6	0.006
EC_e_	25.3	0.006	TOC	19.3	0.032
Na^+^	25.0	0.006	pH-KCl	15.5	NS
Cl^−^	24.2	0.004	C/N	10.5	NS
K^+^	21.0	0.022	P_ca_	10.0	NS
HCO_3_^−^	21.0	0.008			
SP	16.2	NS			
SO_4_^2−^	7.9	NS			
